# Aberrant Phenotype in Human Endothelial Cells of Diabetic Origin: Implications for Saphenous Vein Graft Failure?

**DOI:** 10.1155/2015/409432

**Published:** 2015-04-08

**Authors:** Anna C. Roberts, Jai Gohil, Laura Hudson, Kyle Connolly, Philip Warburton, Rakesh Suman, Peter O'Toole, David J. O'Regan, Neil A. Turner, Kirsten Riches, Karen E. Porter

**Affiliations:** ^1^Division of Cardiovascular and Diabetes Research, Leeds Institute of Cardiovascular and Metabolic Medicine, University of Leeds, Leeds LS2 9JT, UK; ^2^School of Molecular and Cellular Biology, University of Leeds, Leeds LS2 9JT, UK; ^3^Multidisciplinary Cardiovascular Research Centre (MCRC), University of Leeds, Leeds LS2 9JT, UK; ^4^Department of Biology, University of York, York YO10 5DD, UK; ^5^Department of Cardiac Surgery, The Yorkshire Heart Centre, Leeds General Infirmary, Leeds LS1 3EX, UK

## Abstract

Type 2 diabetes (T2DM) confers increased risk of endothelial dysfunction, coronary heart disease, and vulnerability to vein graft failure after bypass grafting, despite glycaemic control. This study explored the concept that endothelial cells (EC) cultured from T2DM and nondiabetic (ND) patients are phenotypically and functionally distinct. Cultured human saphenous vein- (SV-) EC were compared between T2DM and ND patients in parallel. Proliferation, migration, and *in vitro* angiogenesis assays were performed; western blotting was used to quantify phosphorylation of Akt, ERK, and eNOS. The ability of diabetic stimuli (hyperglycaemia, TNF-*α*, and palmitate) to modulate angiogenic potential of ND-EC was also explored. T2DM-EC displayed reduced migration (~30%) and angiogenesis (~40%) compared with ND-EC and a modest, nonsignificant trend to reduced proliferation. Significant inhibition of Akt and eNOS, but not ERK phosphorylation, was observed in T2DM cells. Hyperglycaemia did not modify ND-EC function, but TNF-*α* and palmitate significantly reduced angiogenic capacity (by 27% and 43%, resp.), effects mimicked by Akt inhibition. Aberrancies of EC function may help to explain the increased risk of SV graft failure in T2DM patients. This study highlights the importance of other potentially contributing factors in addition to hyperglycaemia that may inflict injury and long-term dysfunction to the homeostatic capacity of the endothelium.

## 1. Introduction

The prevalence of type 2 diabetes (T2DM) is increasing globally with approximately 3.2 million patients in the UK alone, plus a further 630,000 patients undiagnosed [1]. The leading cause of mortality in such patients is cardiovascular disease (CVD) [2], with T2DM patients suffering a threefold increase in CVD mortality over those without diabetes [3]. Insulin resistance is a feature of prediabetes, a silent condition that is difficult to diagnose early due to compensatory hyperinsulinaemia that can maintain glycaemia and delay diagnosis of the disease. Accordingly, over 30% of newly diagnosed T2DM patients present with cardiovascular complications [4] the treatment of which places a significant burden and inevitable impact on healthcare costs.

Hyperglycaemia is the hallmark of diabetes and clinical trials have revealed that intensive glycaemic control can ameliorate the microvascular complications of T2DM. However, macrovascular complications persist at least in the medium term, particularly in patients with active coronary heart disease [5, 6], suggesting that factors other than glycaemia may contribute to persistent vascular dysfunction. T2DM patients frequently present with a variety of metabolic disturbances such as hyperinsulinaemia, inflammation (elevated inflammatory cytokines such as tumor necrosis factor alpha; TNF-*α*), and disturbed lipid profile (elevated free fatty acids, e.g., palmitate) that may have a cumulative effect and impart persistent pathological changes on EC that are not easy to reverse [7].

The endothelium is a highly metabolic monolayer of cells that lines the luminal surface of all blood vessels and is a principal moderator of vascular health (reviewed in [7]). However, endothelial dysfunction is perceived to precede the development of overt CVD. In health, the endothelium maintains a fine balance of secreted factors that maintain vascular homeostasis. However, in prediabetic patients with insulin resistance this balance is disturbed and favours prothrombotic and vasoconstrictive effects [7].

Endothelial dysfunction predisposes patients to atherosclerosis and coronary artery disease; thus revascularisation procedures are almost a requirement in diabetic patients [8, 9]. The principal method of revascularisation is coronary artery bypass grafting (CABG), frequently performed using the autologous long saphenous vein (SV) to bypass diseased coronary arteries. During SV harvesting, the endothelial layer is damaged; therefore the ability of endothelial cells (EC) to proliferate and migrate is essential to restore homeostasis. Migration of EC is also vital for angiogenesis and the formation of collateral blood vessels, which provide alternate pathways of blood flow in the damaged coronary circulation.

Whilst premature coronary artery disease is evident in T2DM patients, these individuals also suffer inferior outcomes post-CABG [10, 11]. One particular study showed that failure of SV grafts occurred in 9.7% of nondiabetic patients 1-year post-CABG, which increased to 14.4% in the diabetic group [12]. Whilst bypass graft failure is multifactorial, endothelial dysfunction is certainly a key factor. It is likely that the impaired capacity of EC to proliferate and migrate is at least partly due to metabolic disturbances in these patients.

The aims of this study were twofold; firstly, to examine the* in vitro* phenotype and function of SV-EC from patients with or without T2DM, and secondly to investigate the effects of candidate “diabetic” stimuli on nondiabetic (ND) endothelial function.

## 2. Methods

### 2.1. SV-EC Isolation and Culture

Samples of undistended SV were collected from patients undergoing coronary artery bypass grafting at the Leeds General Infirmary. Local ethical committee approval and informed patient consent were obtained. The study conformed to the principles outlined in the Declaration of Helsinki. SV-EC were isolated using Worthington Type II collagenase digestion (1 mg/mL, Lorne Laboratories, Berkshire, UK) as we described previously [13]. Cells were maintained in M199 medium (Sigma-Aldrich, Dorset, UK) supplemented with 20% foetal calf serum (FCS; Labtech International, Sussex, UK), 1% L-glutamine, 1% antibiotic, 20 mM HEPES (all Life Technologies, Paisley, UK), 15 *μ*g/mL endothelial cell growth supplement, 1 *μ*M pyruvate (both Sigma-Aldrich), and 5 U/mL heparin (LEO Laboratories Ltd., Hurley, UK) in a humidified atmosphere of 5% CO_2_ in air at 37°C and passaged using trypsin/EDTA (Life Technologies) as necessary. Endothelial phenotype was confirmed by positive staining for von Willebrand factor. All experiments were performed on early passage cells (p3–7) from patients with (T2DM-EC) or without (ND-EC) diabetes. All T2DM patients were receiving oral therapies alone or supplemented with insulin.

### 2.2. Morphology

Subconfluent SV-EC monolayers were imaged at ×100 magnification and spread cell areas measured from 50 cells per patient population as previously described [14]. In addition, cell circularity was measured using ImageJ software (http://imagej.nih.gov/ij/) and expressed relative to a perfect circle with a value of 1.0.

### 2.3. Proliferation

SV-EC were seeded at a density of 2 × 10^4^ cells per well in 12-well plates and proliferation assays performed essentially as previously described [15, 16]. Following overnight quiescence in minimal medium (MM; containing 1% FCS), cells were reexposed to medium containing 20% FCS (designated “day 0”) for up to 5 days, with medium replenished on day 3. Viable cells were counted on days 0, 3, and 5 using a haemocytometer and trypan blue (Sigma-Aldrich). Further experiments were performed using a range of FCS concentrations (5–20%) and all cell counts made on day 5. Data were expressed as both a percentage increase in cell number (where day 0 counts = 100%) and area under curve (AUC).

To examine SV-EC proliferation/death in real time, time-lapse ptychography [17] was used. Cells were seeded at a density of 7.5 × 10^4^ cells per well in glass-bottomed 6-well plates. After overnight quiescence in MM, cells were reexposed to medium containing 20% FCS for 3 d and maintained in a humidified environment of 5% CO_2_ in air at 37°C in an incubator surrounding a Phase Focus Virtual Lens (PFVL21) microscope. Images were acquired as described previously [18]. Briefly a 20x objective (na 0.4) and a 635 nm illumination were used to obtain a 0.2 mm^2^ region at the centre of the dish. Images were acquired every ~30 min for 3 d and used to create a time-lapse video. Finally, these cells were trypsinised and viability was measured using a Beckman Coulter Vi-CELL.

### 2.4. Scratch Wound Migration

Migration was analysed using a linear scratch wound model essentially as we previously described [15, 16]. Briefly, confluent SV-EC monolayers were quiesced in MM for 24 h, wounded with a sterile 10 mL pipette tip to generate a cell free area, imaged, and treated with medium containing FCS (1–20%) for 24 h after which the number of cells migrated into the wounded area was quantified. Data are represented as percentage increase in migration (versus 1% FCS media (MM) = 100%).

### 2.5. Signalling

Cells were quiesced in MM for 16 hours before preparing whole cell lysates and immunoblotting as previously described [19]. Antibodies were used at the following concentrations: phospho-eNOS (Ser1177, BD Biosciences, 1 : 800), phospho-Akt (Ser473, Cell Signaling Technology, 1 : 500), phospho-ERK1/2 (Thr202 and Tyr204, Cell Signaling Technology, 1 : 800), total Akt (Cell Signaling Technology, 1 : 500), total ERK (Cell Signaling Technology, 1 : 1000), and glyceraldehyde 3-phosphate dehydrogenase (GAPDH; AbCam, 1 : 4000). Films were scanned using an Epson Perfection 4490 flatbed scanner and densitometry analysis performed using ImageJ software (http://imagej.nih.gov/ij/). Data are expressed as a percentage of a “control” lysate (designated as 100%) that was loaded on all membranes to allow comparison between experiments. In additional experiments, cells were stimulated with 100 nM insulin (Sigma-Aldrich) for 5 min and lysates were immunoblotted for p-Akt and p-ERK.

### 2.6. Angiogenesis

*In vitro* angiogenesis was investigated using Matrigel (VWR International, Lutterworth, UK) tube-forming assays. Briefly, 1 × 10^5^ SV-EC were seeded in MM in duplicate wells onto polymerised Matrigel 24-well plates. Plates were incubated at 37°C in a humidified chamber with 5% CO_2_ in air for up to 24 h. The number of intact tubes was counted in ten random ×100 fields by two independent observers. In further experiments, SV-EC were pretreated with either Akt inhibitor LY294002 (1 *μ*M; Merck-Millipore, Watford, UK), eNOS inhibitor (L-N^G^-nitroarginine methyl ester, L-NAME; 500 *μ*M; Sigma-Aldrich), high glucose (25 mM; Sigma-Aldrich), TNF-*α* (1 ng/mL; Life Technologies), palmitate (100 *μ*M; Sigma-Aldrich), or appropriate control vehicle for 24 h prior to angiogenesis assays. Cells were incorporated into assays without replenishment of the stimulus/inhibitor and tube formation was quantified after 8 h.

In selected experiments, time-lapse ptychography [17] was performed in order to visualise any differences in ND and T2DM tube dynamics, for example, the rate of formation or breakdown. In those experiments, cells were plated on Matrigel-coated glass-bottomed 35 mm dishes and maintained in a humidified environment in 5% CO_2_ (in air) at 37°C by an incubator surrounding the PFVL21 microscope. Images were acquired as described previously [18]; briefly a 10x objective (na 0.3) and a 635 nm illumination were used to obtain a 5.88 mm^2^ region at the centre of the dish. Image acquisition began 2 h after seeding of the cells, taken every 10 min for a total period of 24 h and used to create a time-lapse video such that tube formation and degradation could be monitored.

### 2.7. Rhodamine Phalloidin Immunofluorescence

SV-EC were seeded in gelatin-coated chamber slides at a density of 1 × 10^4^ cells per well, cultured for 72 h in complete growth medium then fixed in 4% paraformaldehyde. To determine effects of “diabetic” stimuli, cells were quiesced in MM and then incubated in medium containing 5% FCS, either alone or supplemented with high glucose (25 mM), TNF-*α* (1 ng/mL), palmitate (100 *μ*M), or appropriate controls for 24 h before fixing. The F-actin cytoskeleton was visualised using fluorescent rhodamine phalloidin (Life Technologies) as previously described [20]. All images were captured on a Zeiss Axio Imager Z1 epifluorescence microscope at ×200 and ×400 magnification.

### 2.8. Statistics

All data are expressed as mean ± SEM, with *n* representing the number of experiments on cells from different patients. Data were analysed using standard or ratio (log transformed) two-way ANOVA or Mann-Whitney *t*-test as appropriate, using GraphPad software (www.graphpad.com), with *P* < 0.05 considered statistically significant.

## 3. Results

### 3.1. Population Demographics

Experiments were performed on endothelial cells from a total of 44 patients. The mean age of ND and T2DM patients was not significantly different: ND (*n* = 27, 85% male), 64.5 ± 1.6 (range 50–80) years versus T2DM (*n* = 17, 82% male), and 67.0 ± 2.1 (range 48–78) years; *P* = 0.35. All T2DM patients were receiving oral therapy (metformin/sulfonylureas/gliptins), and 30% of these were also receiving insulin. Routine cardiovascular medications (statins, *β*-blockers, angiotensin modulating agents, anticoagulation therapies, and diuretics) were similar in both cohorts as we have reported previously [21].

### 3.2. SV-EC Morphology

**Figure 1:**
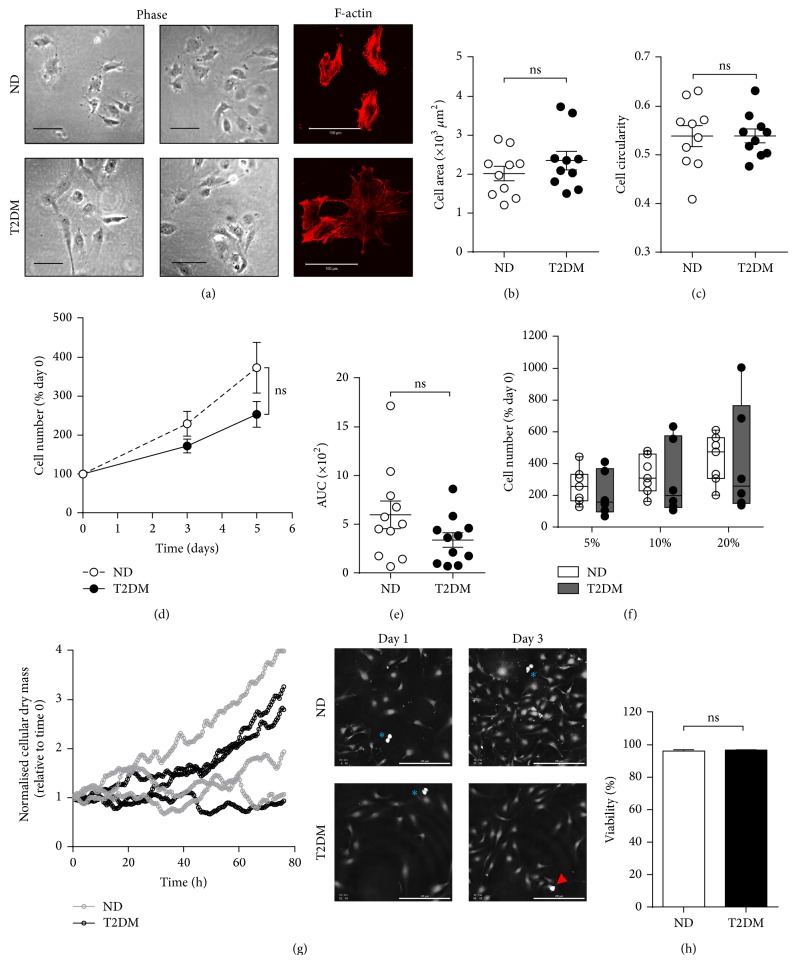
SV-EC morphology and proliferation. (a) Morphology of ND and T2DM populations in culture, scale bar = 100 *μ*m. Left-hand panels = representative phase images from two ND and two T2DM-EC, right-hand panels = rhodamine phalloidin staining of F-actin cytoskeleton at ×400 magnification from one ND and one T2DM-EC population. (b) Spread cell area and (c) mean circularity (roundness) of 50 cells per population of ND and T2DM-EC were measured using ImageJ (both *n* = 10, ns = not significant). (d) Proliferation was quantified by direct counting of live cells over 5 days in full growth medium. Data are expressed as the percentage increase in cell number from day 0 count and (e) area under curve (AUC) (*n* = 11, ns = not significant). (f) SV-EC were treated with 5–20% FCS and viable cells counted on day 5. Data is presented as box-and-whiskers with the median indicated (*n* = 6-7, ns = not significant). (g) Cells from three ND and three T2DM donors were monitored over a period of 3 days in full growth medium on a Phase Focus PFVL21 system and the relative cellular dry mass plotted over time. Right-hand panel = representative ptychographic images of cells dividing (blue asterisks) and dying (red arrowhead), scale bar = 200 *μ*m. (h) Cell viability following completion of the ptychographic experiment, *n* = 3, ns = not significant.

In contrast to the distinct morphological and proliferative changes we have previously observed in SV smooth muscle cells (SV-SMC) from T2DM patients [14, 21, 22], there were no distinct differences in morphology between ND and T2DM-EC, with all cells exhibiting a typical EC cobblestone appearance and no consistent difference in F-actin organisation (Figure 1(a)). Whilst there was inherent variability in cell size between patients, this was not associated with T2DM (Figure 1(b)). Neither did “circularity” vary between the two populations (Figure 1(c)).

### 3.3. SV-EC Proliferation

Following SV bypass grafting, denuded endothelium regenerates through proliferation and migration. Proliferation was monitored by constructing growth curves over a 5-day period. Although cell number of T2DM EC was ~70% of ND-EC cell number at day 5, this did not reach statistical significance (Figure 1(d)). Area under curve analysis for each cell population, whilst suggesting a trend towards reduced proliferation in the T2DM cells, did not reach statistical significance (Figure 1(e)). A similar degree of T2DM-EC proliferative impairment was visible across a range of concentrations of FCS (Figure 1(f)).

Ptychography [17] is a novel label-free methodology that generates high quality/contrast phase images of living cells with no invasive labelling protocols or requirement for high energy illumination. The ability to digitally focus images after acquisition prevents loss of data due to focal drift of the microscope [18]. ND and T2DM-EC were monitored for 3 d and a quantitative parameter related to cellular dry mass was calculated at each time point as a measure of proliferation. Growth profiles of the 3 cell populations for both ND and T2DM cells over time are represented in Figure 1(g) (left panel). Video data (Suppl. Files 1 and 2 in Supplementary Material available online at http://dx.doi.org/10.1155/2015/409432) show that any reduction in dry mass was due to cells moving out of the field of view and not to increased cell death. Two populations (one each of ND and T2DM-EC) did not proliferate readily, highlighting the inherent interpatient variability as shown by direct cell counting (Figures 1(d)–1(f)). Importantly, the viability of all cell populations was similar between ND and T2DM patients (96.2% and 96.8%, resp.; Figure 1(h)) suggesting that the trend towards reduced proliferation in T2DM-EC was not a result of increased cell death. Indeed, time-lapse imaging showed multiple proliferation events and a lack of cell death events (Figure 1(g), right panel and Suppl. Files 1 and 2).

### 3.4. SV-EC Migration

**Figure 2:**
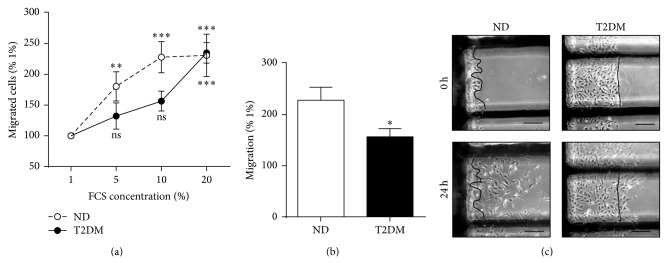
SV-EC migration. Confluent monolayers were quiesced and then wounded to create a cell-free area. Cells were cultured in medium containing 1–20% FCS and images taken at 0 and 24 h. The number of cells migrated past the wound edge were counted. (a) Concentration response to FCS, expressed relative to migration observed in 1% FCS (*n* = 9, ^∗∗∗^ *P* < 0.001, ^∗∗^ *P* < 0.01, ns = not significant relative to 1% migration). (b) Summary data of migration in media containing 10% FCS (*n* = 9, ^∗^ *P* < 0.05). (c) Representative images, scale bar = 100 *μ*m. Cells can clearly be seen migrating past the wound edge at 24 h.

EC motility was assessed using a scratch wound model where SV-EC migration into a denuded area over 24 h (Figure 2(c)) was evaluated. FCS-induced migration in both ND and T2DM-EC was concentration dependent, although the response in T2DM-EC was blunted (Figure 2(a)). Accordingly, in 10% FCS, T2DM-EC migration was reduced by >30% compared to ND-EC (Figures 2(b) and 2(c)).

### 3.5. SV-EC Signalling

**Figure 3:**
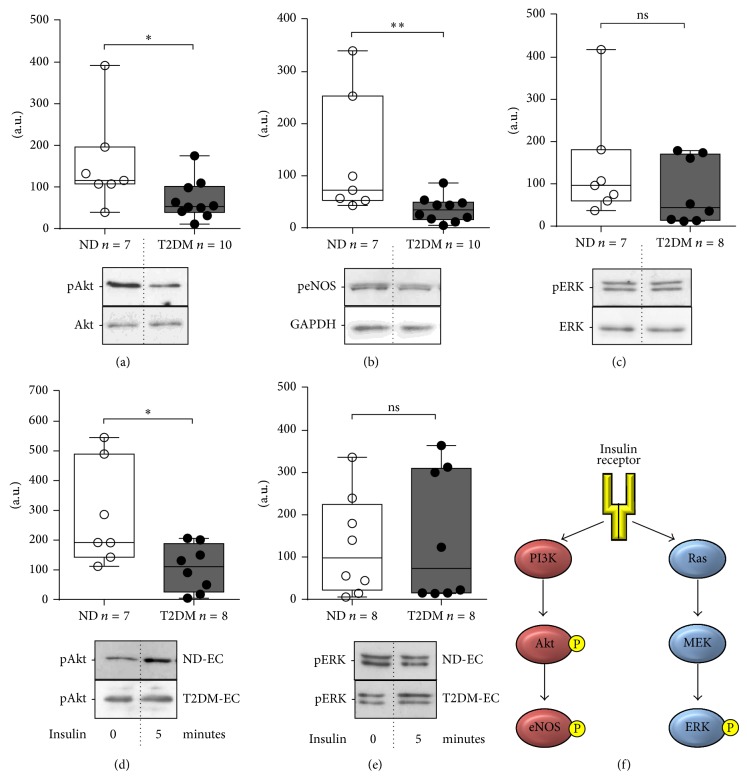
Intracellular signalling in SV-EC. Cells were quiesced in MM (1% FCS) for 16 h and cell lysates immunoblotted for basal phosphorylation of (a) Akt, (b) eNOS, and (c) ERK. Data was normalised to a control lysate included on every blot and presented as box-and-whiskers with the median line indicated (upper panel), ^∗∗^ *P* < 0.01, ^∗^ *P* < 0.05, ns = not significant. Lower panels = representative western blots. Sensitivity to insulin was determined by treating quiesced cells with 100 nM insulin for 5 min. Cell lysates were immunoblotted for phosphorylation of (d) Akt and (e) ERK; ^∗^ *P* < 0.05, ns = not significant. Lower panels = representative western blots showing basal phosphorylation and in response to insulin. (f) Simplified diagram of the insulin signalling pathway. P = phosphorylation.

It is generally perceived that aberrancies of intracellular signalling pathways impact on cellular function. We therefore explored the concept that basal differences in two principal insulin-stimulated signalling pathways, Akt and ERK (Figure 3(f)), may be evident between ND and T2DM SV-EC. Cells were cultured in MM and phosphorylation of Akt, eNOS, and ERK quantified using western blotting. Basal phosphorylation states of both Akt (Figure 3(a)) and eNOS (Figure 3(b)) were significantly lower in T2DM-EC (56% and 73% of ND-EC, resp.). In contrast, phosphorylation of ERK was similar in each population (Figure 3(c)). Diminished Akt phosphorylation in T2DM cells was maintained in response to stimulation with insulin (Figure 3(d)) whilst levels of ERK phosphorylation were similar between populations (Figure 3(e)).

### 3.6. SV-EC* In Vitro* Angiogenesis

**Figure 4:**
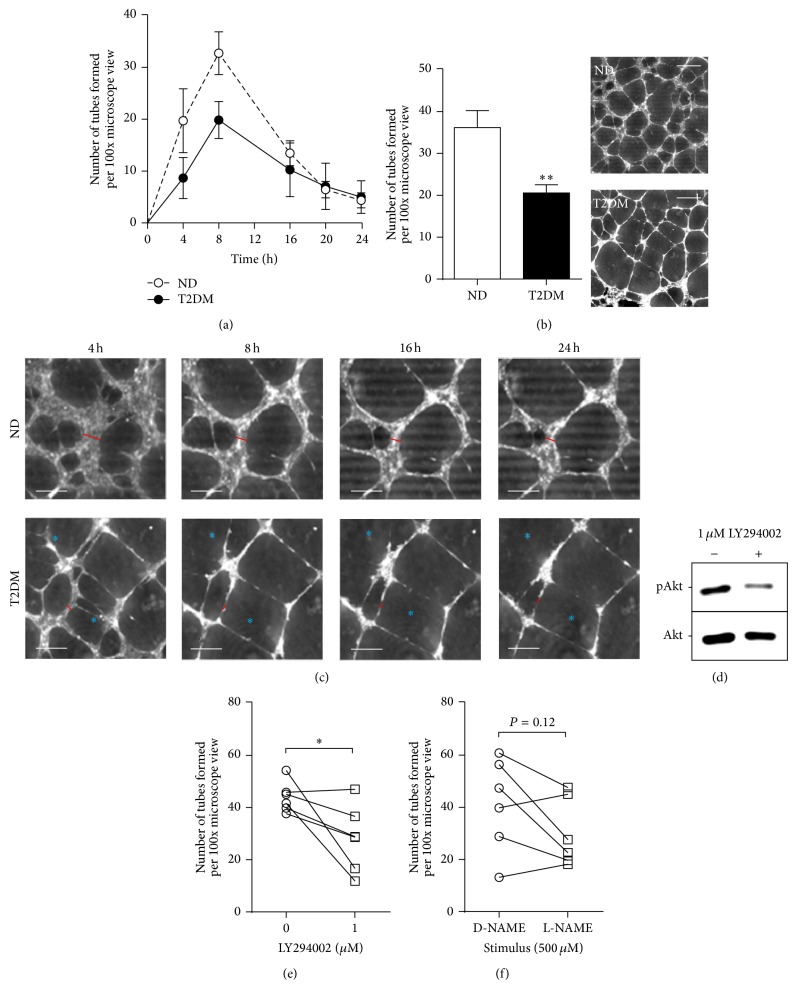
SV-EC angiogenesis. Angiogenesis assays were performed by seeding EC onto Matrigel and quantifying the number of tubes formed over time. (a) The number of intact tubes in 10 fields per population (×100 magnification) were quantified over 4–24 h by two independent observers (*n* = 4). (b) Number of intact tubes formed after 8 h (*n* = 7, ^∗∗^ *P* < 0.01) with representative images using the Phase Focus Virtual Lens (PFVL21). Scale bar = 500 *μ*m. (c) Enlarged images showing the same field over extended time course. The width of tube walls is indicated by a red bar, and breakage of walls is indicated by blue asterisks (scale bar = 250 *μ*m). (d) Cells were exposed to 1 *μ*M LY294002 for 24 h and lysates were immunoblotted for phosphorylation of Akt. (e) ND-EC were treated with 1 *μ*M LY294002 for 24 h prior to incorporation into angiogenesis assays and the number of intact tubes counted (*n* = 5, ^∗^ *P* < 0.05). (f) Similar experiments were performed after pretreatment with 500 *μ*M L-NAME or control (D-NAME) (*n* = 5, *P* = 0.12).

Patients with T2DM have impaired ability to form collateral blood vessels [23]; it is likely that the reduced migratory capacity is a factor contributing to inferior angiogenesis. Using* in vitro* tube-forming assays, time courses were performed, revealing that the temporal profile of tube formation was similar in ND-EC and T2DM-EC. However, whilst each SV-EC population exhibited a similar temporal profile of tube formation which was maximal after 8 h, the number of tubes was significantly fewer in the T2DM group (~60% ND) (Figures 4(a) and 4(b)).

To explore the kinetics of tube formation, we performed time-lapse ptychography over a 24 h interval. The establishment of tube-like networks was evident in both ND-EC and T2DM-EC after 2 h, the earliest time point at which the technique could detect all cells within the same plane. In ND-EC, tubes gradually expanded over the 24 h and formed stable, robust walls comprised of multiple cell layers (denoted by red bars in Figure 4(c); also see Suppl. File 3). In T2DM-EC tubes appeared to expand more rapidly than ND-EC (Suppl. File 4) but tube walls appeared fragile (generally only one cell thick) and there were frequent broken/incomplete tubes (illustrated by asterisks in Figure 4(c)).

T2DM-EC exhibited marked reduction in basal levels of Akt and eNOS phosphorylation (Figure 3). To determine whether inhibiting Akt or eNOS activity modulated* in vitro* angiogenesis, we treated ND-EC with appropriate inhibitors (LY294002 and L-NAME, resp.) prior to tube-forming assays. Exposure to 1 *μ*M LY294002 for 24 h resulted in a reduction in Akt phosphorylation confirming the efficacy of the inhibitor in these experiments (Figure 4(d)). Akt inhibition significantly impaired tube forming by 36% (Figure 4(e)); however the effect of L-NAME was inconsistent between cells from different patients (Figure 4(e)).

### 3.7. Effects of “Diabetic” Stimuli

**Figure 5:**
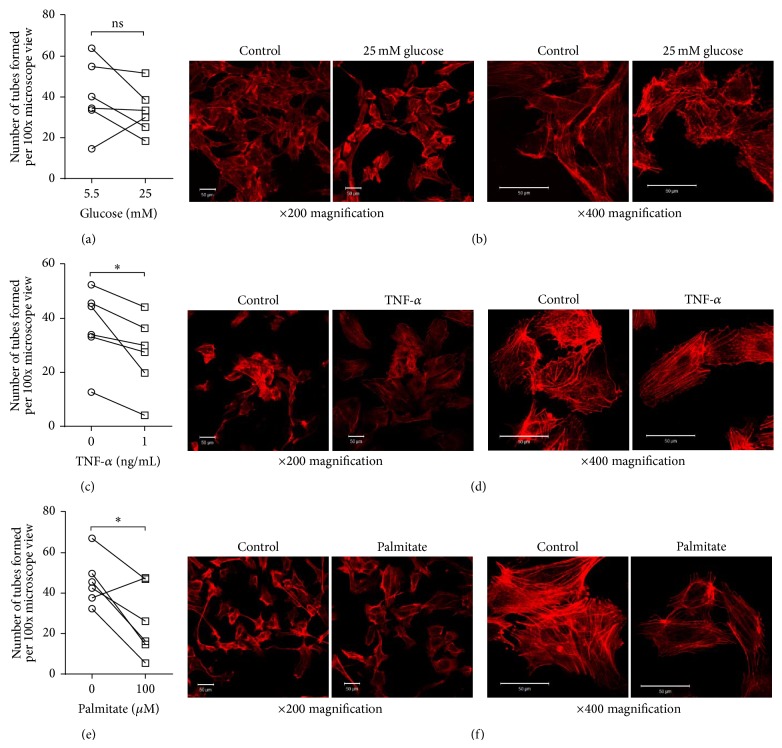
Effect of “diabetic” stimuli on SV-EC function. (a) ND-EC were treated with 25 mM glucose (with mannitol as osmolarity control) for 24 h before performing angiogenesis assays and counting the number of intact tubes (*n* = 5). (b) Similarly treated cells were fixed and the F-actin cytoskeleton visualised using rhodamine phalloidin. Scale bars = 50 *μ*m. (c) Parallel assays were performed on cells treated with 1 ng/mL TNF-*α* as an inflammatory stimulus ((c, d), *n* = 6, ^∗^ *P* < 0.05) and 100 *μ*M palmitate to mimic an elevation in free fatty acids ((e, f), *n* = 6, ^∗^ *P* < 0.05).

ND-EC were cultured under conditions designed to mimic the* in vivo* environment of T2DM and the impact on angiogenesis assays was evaluated. We also examined the F-actin cytoskeleton due to its important role in migration and intercellular communication [24]. Surprisingly, 25 mM glucose had no impact on tube formation or the cytoskeleton (Figures 5(a) and 5(b)). In contrast, both the proinflammatory cytokine TNF-*α* and free fatty acid palmitate significantly impaired angiogenesis, by 27% and 43%, respectively (Figures 5(c) and 5(e)). There was reduced F-actin staining intensity and reorganisation from peripheral fibres in control cells to more centrally aligned stress fibres in the TNF-*α*-treated cells (Figure 5(d)). In palmitate treated cells a similar reduction in staining intensity was observed and although peripheral localisation was maintained, fibres in the body of the cell appeared more disorganised (Figure 5(f)).

## 4. Discussion

A key strength of this study was the use of the exact cells whose dysfunction underlies failure or complications following bypass grafting in T2DM patients, namely, SV-EC. During harvesting and grafting for CABG the endothelium is inevitably denuded and hence EC regeneration through both proliferation and migration is pivotal to restore a functional monolayer. In contrast to SV-SMC [14, 21, 22], gross SV-EC morphology was indistinguishable between ND and T2DM patients; although this does not rule out the possibility of subcellular alterations. Instead, we focused on differences in cellular function that would be viewed as key to cardiovascular health. Importantly, vital functions were persistently impaired, namely, migration, tube forming, and, to a lesser degree, proliferation. Lehle et al. previously described a significant inhibition of proliferation in T2DM-EC isolated from human SV [25]. Indeed, the difference between the ND and T2DM cells in our study appears more distinct at later time points and extending the study beyond 5 days would potentially answer this question. Importantly, the modest reduction in T2DM-EC proliferation was not a result of increased cell death.

EC migration is also an essential process in reendothelialisation. Indeed, previous studies have demonstrated impaired migration in bovine [26] and human aortic EC [27] under conditions of hyperglycaemia, suggesting that the diabetic milieu may harm the migratory capacity of resident cells. Studies in a murine model of T2DM also reported impaired EC migration in the* db/db* mouse [28]. In human studies, reduced migration of endothelial progenitor cells from T2DM patients was impaired relative to ND patients [28]. Accordingly we observed a significant impairment in migration of native T2DM-EC relative to ND-EC. Importantly this divergence was observed under normoglycaemic conditions (5.5 mM glucose), lending credence to the notion of a persistent EC phenotype imprinted* in vivo* and that does not reverse easily in culture.

Insulin signals through the insulin receptor via two main arms, triggering the Akt and ERK pathways [29] (Figure 3(f)). It is perceived that in T2DM patients the Akt pathway is impaired with signalling preferentially stimulating the ERK pathway [7]. To our knowledge this is the first direct demonstration of divergent Akt and ERK signalling, both basally and following insulin stimulation, in human SV-EC, another feature maintained in culture. This is in agreement with evidence in animal models of diabetes [30] and human skeletal muscle [31]. Since activation of Akt leads to eNOS phosphorylation (Figure 3(f)), it is perhaps not surprising that eNOS phosphorylation was also diminished in T2DM-EC. Literary evidence for differential eNOS phosphorylation in T2DM is disparate. Previous studies have revealed reduced Akt and eNOS phosphorylation in atrial biopsies from T2DM patients [32] which concurs with the data presented here; however phosphorylation of eNOS was reportedly elevated in venous EC from T2DM patients' forearms [33]. This highlights the importance of evaluating the strengths and weaknesses of different experimental models and advocates the use of an appropriate cellular model (i.e., SV-EC to explore dysfunction with respect to graft failure).

An alternative compensatory pathway of restoring blood flow in arteries diseased by atherosclerosis is the formation of collateral blood vessels through angiogenic mechanisms. These small, new vessels help to revascularise the myocardium and bypass the occlusion [34]. Given the increased prevalence of CVD in T2DM patients this is an important mechanism; however it is well recognised that such patients suffer from impaired coronary collateral formation [23] which in turn increases the need for coronary revascularisation. Animal models of diabetes exhibit reduced angiogenic capacity* in vivo* [35, 36]. In our study we used a well validated* in vitro* model to study SV-EC angiogenesis and believe this is the first demonstration of impaired angiogenic capacity in human T2DM-EC versus ND-EC. Ptychography was used to monitor tube formation dynamics without damaging or altering the cells, for example, by using fluorescent labels that potentially interfere with cell behaviour. This revealed that the initial establishment of tubes was not different between ND and T2DM-EC; however T2DM-EC tubes expanded rapidly once established and exhibited thin, fragile walls that started to degenerate after 8 h. Combining this knowledge with the reduced total number of tubes observed at 8 h, it is likely that T2DM-EC tubes are less stable than ND-EC. Ptychography may be a useful technique to distinguish between the absolute capacity to form and maintain robust tubes and the ability to monitor degeneration. Moreover, the absence of a requirement for cell “labelling” of any kind, together with a diffuse, low-powered 635 nm illumination, provides confidence that the cell has not been modified by such markers.

Having observed reduced Akt and eNOS phosphorylation in native T2DM-EC, we explored whether Akt and/or eNOS inhibition impaired angiogenesis in EC of nondiabetic origin. Whilst Akt inhibition significantly reduced tube formation, the response to eNOS inhibition was inconsistent; four out of six cell populations showing reduced tube formation to varying degrees. Other downstream effectors of Akt, for example, vascular endothelial growth factor [37], may additionally be necessary in order to elicit a significant and robust inhibition.

Finally, we explored the effects of candidate “diabetic” stimuli on* in vitro* angiogenesis. Hyperglycaemia reportedly impairs EC migration [26, 27] and inhibits angiogenesis in human microvascular EC [38] and HUVEC [39]. Somewhat unexpectedly we observed no effect of consistent high glucose on angiogenesis in SV-EC, possibly reflecting differences in the sources of EC. Microvascular and macrovascular EC are phenotypically distinct [40], and HUVECs express a foetal gene pattern which is divergent from SV-EC [41]. Another possible explanation is that variability in glycaemic control is more deleterious to endothelial cells than persistent hyperglycaemia. For example, some previous studies reported that fluctuating or intermittent high glucose increased expression of adhesion molecules ICAM-1, VCAM-1, and E-selectin [42], augmented apoptosis [43], and downregulated NO production [44]. No studies have investigated hyperglycaemia* per se* on human SV-EC angiogenesis but the failure of intensive glycaemic control to normalise macrovascular complications in T2DM in the medium term (ACCORD, ADVANCE, and VADT clinical trials [5, 6]) suggests involvement of other factors.

Circulating levels of TNF-*α* are elevated in T2DM patients [45] but reports of its effects on angiogenesis are contradictory. TNF-*α* inhibits tube-forming* in vitro* [46] yet paradoxically increases angiogenesis* in vivo* [47]. Duration of exposure to TNF-*α* is reportedly key to its pro- or antiangiogenic effects [48], and dysfunctional angiogenesis has been attributed to attenuated eNOS activity [49] and modification of Rho GTPase activity [50]; the latter potentially associated with disturbed F-actin dynamics as observed in the present study and previously noted by others [51].

Raised plasma levels of free fatty acids are associated with metabolic syndrome [52]. Palmitate is proapoptotic to EC [53, 54] although its effect on angiogenesis is not clear. In our study,* in vitro* tube-forming was markedly impaired with comparatively low (100 *μ*M) concentrations of palmitate and could be attributable to inhibition of eNOS phosphorylation [53]. Our data also indicate reduced staining intensity of F-actin and disorganisation of identifiable fibres in palmitate-treated cells. Palmitate was shown to reduce F-actin content in L6 myotubes, reportedly via accumulation of membrane cholesterol [55]; it is therefore possible that this mechanism exists in SV-EC.

The “diabetic” stimuli we chose to employ in this study are neither exclusive nor exhaustive. Multiple cytokines and chemokines relevant to diabetic complications are induced by hyperglycaemia, including interleukin-1 beta (IL-1*β*) [56]. Indeed, hyperglycaemia-induced IL-1*β* expression in retinal endothelial cells is believed to underlie diabetic retinopathy [57]. Interestingly, a very recent study demonstrated that monocytes exposed to palmitate secreted IL-1*β*, which in turn, induced EC adhesion molecule expression, an effect abolished by IL-1 receptor antagonism [58]. IL-1*β* clearly plays an important role, both directly and indirectly via effects on other pathways.

The maintenance of a persistent aberrant T2DM-EC phenotype under culture conditions is perhaps indicative of the phenomenon of metabolic memory [59]; that is, the concept that prior exposure to hyperglycaemia and/or other metabolic insults* in vivo* can lead to maintained cellular effects* in vitro*. This is likely to involve epigenetic mechanisms such as microRNAs, histone modifications and/or DNA methylation (reviewed in [7]). Indeed, transient exposure to hyperglycaemia in human and bovine EC imparted persistent changes in the nuclear factor kappa B (NF*κ*B) signalling pathway that were retained even when cells were returned to normoglycaemia [60, 61]. MicroRNAs (miRs) are short, noncoding RNAs that negatively regulate target gene expression and have been proposed as biomarkers for cardiovascular disease and diabetes [62]. For example, miR-21, widely expressed in the vasculature, has been reported to inhibit proliferation, migration, and angiogenesis in HUVEC [63]. It is entirely possible that similar mechanisms exist in human SV-EC and studies to investigate this hypothesis are currently in progress.

## 5. Conclusions

To our knowledge, this study is the first to demonstrate divergent migration and tube forming capacity in SV-EC cultured from patients with and without T2DM. There was a nonsignificant trend towards impaired proliferation. Importantly we provide direct evidence for selective impairment of the Akt, but not ERK signalling pathway in these clinically relevant cells. This T2DM phenotype persisted throughout culture and passaging, suggestive of epigenetic changes inflicted* in vivo* and not easily reversed. ND-EC exposed to diabetic stimuli adopted a T2DM-EC phenotype, suggesting the importance of factors other than hyperglycaemia in promoting endothelial dysfunction in T2DM. In conclusion, the blunted functionality of SV-EC from T2DM patients in our study, together with our demonstration that diabetic stimuli can impair aspects of ND-EC behavior, may translate into an inadequate or delayed ability to reendothelialise, significantly impacting the homeostatic capabilities of the endothelium and predisposing to graft failure.

## Supplementary Material

Supplementary File 1: ND-EC proliferation. Ptychographic time-lapse video of ND-EC over 3 days, showing clear evidence of cell division (proliferation) over the course of the assay.
Supplementary File 2: T2DM-EC proliferation. Ptychographic time-lapse video of T2DM-EC over 3 days. In the final frames, only one apoptotic cell was visible (lower right-hand side of the frame).
Supplementary File 3: ND-EC angiogenesis. Ptychographic time-lapse video of ND-EC tube formation. Recording commenced 2 h post-plating and video was collated from hourly images. Tubes with robust walls were observed forming and expanding over the course of the assay.
Supplementary File 4: T2DM-EC angiogenesis. Ptychographic time-lapse video of T2DM-EC tube formation. Recording commenced 2 h post-plating and video was collated from hourly images. Whilst tubes formed and rapidly expanded over the course of the assay, tube walls appeared narrower than those observed in ND-EC.
